# Role of antioxidants supplementation in the treatment of atopic dermatitis: a critical narrative review

**DOI:** 10.3389/fnut.2024.1393673

**Published:** 2024-06-12

**Authors:** Edoardo De Simoni, Matteo Candelora, Sara Belleggia, Giulio Rizzetto, Elisa Molinelli, Irene Capodaglio, Gianna Ferretti, Tiziana Bacchetti, Annamaria Offidani, Oriana Simonetti

**Affiliations:** ^1^Clinic of Dermatology, Department of Clinical and Molecular Sciences, Polytechnic University of Marche, Ancona, Italy; ^2^Hospital Cardiology and UTIC, Ospedali Riuniti di Ancona, Ancona, Italy; ^3^Department of Clinical Experimental Science and Odontostomatology-Biochemistry, Research Center of Health Education and Health Promotion, Ancona, Italy; ^4^Department of Life and Environmental Sciences-Biochemistry, Polytechnic University of Marche, Ancona, Italy

**Keywords:** atopic dermatitis, atopic eczema, oxidative stress, reactive oxygen species, exogenous antioxidants, antioxidant supplementation, treatment

## Abstract

Atopic dermatitis (AD) is a chronic inflammatory skin disease characterized by itching, epidermal barrier dysfunction, and an unbalanced inflammatory reaction. AD pathophysiology involves a dysregulated immune response driven by T helper-2 cells. Many factors, including reactive oxygen species (ROS), are involved in AD pathogenesis by causing cellular damage and inflammation resulting in skin barrier dysfunction. This narrative review aims to provide a comprehensive overview of the role of natural molecules and antioxidant compounds, highlighting their potential therapeutic value in AD prevention and management. They include vitamin D, vitamin E, pyridoxine, Vitamin C, carotenoids, and melatonin. Some studies report a statistically significant association between antioxidant levels and improvement in AD, however, there are conflicting results in which antioxidant supplementation, especially Vitamin D, did not result in improvement in AD. Therefore, the clinical efficacy of these dietary nutritional factors in the treatment of AD needs to be further evaluated in clinical trials. Meanwhile, antioxidants can be incorporated into the management of AD patients in a personalized manner, tailored to the severity of the disease, comorbidities, and individual needs.

## 1 Introduction

Atopic dermatitis (AD) is a chronic and relapsing inflammatory skin disorder with a multifactorial etiology. It is characterized by itching and has a typical age-dependent distribution of lesions (1). The prevalence of AD ranges from 10–20% in childhood to 2–8% in adulthood and the main manifestations are itching and eczematous lesions in particularly in the flexural areas (1–3). The treatment landscape of AD includes steroids, immunosuppressive agents and biological therapies such as monoclonal antibodies and Jak-inhibitors (4–6).

Most cases of AD are mild, while less than 10% of patients are severely affected. Therefore, the severity of AD is classified from mild to severe according to various clinical scales such as the Eczema Area and Severity Index (EASI), the Investigator Global Evaluation (IGA), the Three Item Severity Score (TIS), and the SCORing Atopic Dermatitis (SCORAD) scales (7). AD usually develops before the age of five, and symptoms persist into adulthood in about 50% of individuals; in addition, early onset of AD is associated with a more severe course of the disease (1, 8).

AD is associated with several comorbidities, both allergic and non-allergic, indicating its systemic nature, particularly bacterial and fungal infections, neuropsychiatric disorders, autoimmune diseases, metabolic diseases and haematologic malignancies (9–14). The association with autoimmune diseases increases with the severity of AD, particularly for conditions such as psoriatic arthritis, alopecia areata, and vitiligo (15, 16).

AD can be distinguished into extrinsic and intrinsic. The extrinsic form is the most common and represents 80% of cases and correlates with elevated serum IgE levels and skin barrier damage. In contrast, the intrinsic form, which is less common, has normal levels of IgE and an intact skin barrier. Several clinical studies have demonstrated the extreme importance of barrier integrity in AD, which can be altered by both genetic and environmental factors. The latter, through the formation of ROS, cause direct membrane damage triggering the genesis of a vicious circle inflammatory process that underlies the pathogenesis of AD (17).

The pathophysiology of AD is complex, combining genetic predisposition and impaired epidermal barrier function with an unbalanced inflammatory response to environmental factors (1, 18). Genetic factors play an important role in AD, involving several genes encoding epidermal structural proteins and elements of the immune system being implicated. Barrier dysfunction in AD patients results from reduced expression of the filaggrin gene, ceramide deficiency and overexpression of epidermal proteases (18–20). The exact primary cause of skin inflammation in AD is poorly understood. However, scratching of the skin may trigger the immune system resulting in activation and release of pro-inflammatory cytokines by keratinocytes (21). Environmental factors, such as infections, pollutants, smoke and detergents, can alter the skin barrier and trigger an inflammatory response through the generation of reactive oxygen species (ROS) (19–22).

The high production of ROS can induce autophagy, apoptosis and programmed necrosis in cells, resulting in DNA damage and skin barrier breakdown (22). Although these reactive species participate in the pathophysiology of AD, the role of phytochemicals and antioxidant agents in preventing or controlling oxidative stress in AD patients has not been fully investigated (23, 24). ROS production also plays a role in other inflammatory diseases including psoriasis. Indeed, increased levels of proinflammatory cytokines, including IL-6, IL-1β, IL-17A, and IL-22, are observed in psoriasis, leading to excessive ROS generation from both endogenous and exogenous sources, resulting in increased oxidative stress. Increased levels of protein oxidation markers, malondialdehyde (MDA), serum nitric oxide and related products correlate with disease severity. This may be partly due to lower total antioxidant activity in these patients, particularly the decrease in SOD and CAT activities (24, 25).

The aim of this narrative review is to summarize the latest evidence on the role of ROS and oxidative stress in the development of AD and to provide an overview of the studies on the effects of exogenous antioxidants in prevention and treatment of patients with AD.

## 2 Materials and methods

We searched PubMed, Medline and Google Scholar for articles published from March 2006 to April 2023 using the following keywords: ‘atopic dermatitis, atopic eczema, oxidative stress, reactive oxygen species, exogenous antioxidants, antioxidant supplementation, treatment’.

We then read the abstract of each article. The entire article was read only if the abstract indicated that the article potentially met our inclusion criteria: English language, research papers, and studies on human population only.

Observational studies, randomized clinical trials (RCTs) and meta-analyses, in English language, were included in this narrative review. The aim of this review is to summarize the most recent evidence on the activity and efficacy of exogenous antioxidants in patients with AD. Research results were reported in Table 1.

**Table 1:** The most important clinical studies with exogenous antioxidants in patients with AD.

Authors and year	Study design and population	Results
Vitamin D		
Amestejani et al. 2012 (26)	60 pts. with AD; 30 treated with Vitamin D 1600 UI and 30 with plb for 60 d.	Improvement in SCORAD and TIS in pts. taking Vitamin D (*p* < 0.05).
Hata et al. 2014 (27)	30 pts. with AD, 30 no-AD, and 16 pts. with psoriasis received Vitamin D 4000 IU or plb for 21 d.	At time 0, 20% of AD pts. had serum Vitamin D < 20 ng/mL but no correlation with AD severity. After 21 days increased serum Vitamin D levels, but no significant changes in EASI.
Galli et al. 2015 (28)	89 children with AD; 1 group received daily oral Vitamin D (2000 IUs) for 3 mos; control group no supplementation.	Vitamin D supplementation did not influence the SCORAD severity (*p* < 0.55).
Di Filippo et al. 2015 (29)	39 children with AD and 20 healthy controls. Pts were treated with Vitamin D oral supplementation of 1,000 IU/day for 3 mos.	Vitamin D supplementation reduced AD severity in children (*p* < 0.001).
Sanchez-Armendariz et al. 2018 (30)	65 pts. with AD. Vitamin D group (*n* = 33) at dose 5,000 IU/day or plb (*n* = 32).	A reduction in the severity of SCORAD when plasma levels of Vitamin D > 20 ng/mL were achieved (*p* = 0.03).
Lama Corrales et al. 2019 (31)	44 pts. with <72.7 nmol/L Vitamin D levels were randomized to either Vitamin D supplementation of 2000 IU/d or plb.	Vitamin D supplementation did not significantly improve disease severity (*p* = 0.7).
Mansour et al. 2020 (32)	92 pts. with AD were randomized to receive either Vitamin D 1600 IU/d or plb for 12 wks.	EASI differed significantly between the supplementation (56.44 ± 29.33) and plb (42.09 ± 19.22) groups after intervention (*p* = 0.039).
El-Heis et al. 2022 (33)	703 mothers; 1,000 IU of Vitamin D/plb die from the 14th week of gestation until delivery were administered. Offspring AD at 12, 24 and 48 mos of age was examined.	At 12 months AD prevalence was lower (OR 0.55; *p* = 0.04); this effect attenuated and was not statistically significant at 24 months (OR 0.76) or 48 months (OR 0.75).
Cabalín et al. 2022 (34)	22 children with AD received weekly oral Vitamin D for 6 wks.	Vitamin D supplementation was associated with SCORAD decreased (*p* < 0.0001).
Javanbakht et al. 2011 (35)	45 AD pts. Group P (*n* = 11): plb Vitamin E and D; Group D (*n* = 12): 1600 IU Vitamin D and Vitamin E plb; Group E (*n* = 11): 600 IU synthetic Vitamin E plus Vitamin D plb; Group DE (*n* = 11), 1,600 IU Vitamin D plus 600 IU synthetic Vitamin E for 60 ds	Increased plasmatic levels of Vitamin E are associated with decreased SCORAD, intensity, objective, and extension (*p* = 0.02)
Vitamin E		
Lee et al. 2012 (36)	119 children aged 0–24 mos	Serum Vitamin E levels were the higher the lower the total and specific IgE (*p* < 0.05).
Jaffary et al. 2015 (37)	70 pts., two groups 35 pts., receiving Vitamin E (400 IU/day) and plb for 4 mos	The improvement in all symptoms, except sleeplessness, was significantly higher in the group receiving Vitamin E than in controls (−1.5 vs. 0.218 in itching, −10.85 vs. –3.54 in extent of lesion, and − 11.12 vs. –3.89 in SCORAD, respectively, *p* < 0.05).
Pyridoxine		
Mabin et al. 2006 (38)	48 AD pts., 19 received 50 mg/day of pyridoxine hydrochloride vs. 22 plb	The two groups show no significant differences
Melatonin		
Yung-Sen Chang et al. 2016 (39)	73 AD pts. with at least 5% skin involvement (1–18 years). Melatonin 3 mg/day vs. plb for 4 wks with a washout of 2 wks	After the intake of melatonin, SCORAD was reduced by 9.1 compared with plb, from 49.1 to 40.2.
Taghavi Ardakani et al. 2018 (40)	70 pts. (6–12 years) with AD. Two groups: 6 mg/ds melatonin supplements vs. plb (*n* = 35 each group) for 6 wks.	Melatonin supplementation significantly improved SCORAD.
Beta-carotene		
Xiang et al. 2019 (41)	81 with AD pts. and 65 healthy individuals.	Serum Vitamin A and Vitamin D are lower in AD children (*p* < 0.001, *p* = 0.0423). SCORAD significantly higher in AD patients with Vitamin A and Vitamin D co-deficiency.
Lycopene		
Inoue et al. 2023 (42)	263 pts. (mothers and children), 40 with AD and 263 with no-AD.	Low lycopene intake during pregnancy correlated with higher risk for infantile AD. Infant blood lycopene level (OR, 0.01; *p* = 0.007) was significantly related as protective to AD.

AD, Atopic dermatitis; CSS, Cross-sectional study; d, day; EASI, Eczema Area and Severity Index; FTS, Fitzpatrick Skin Type; HBD, human beta-defensin; IgE, Immunoglobulin E; mos, Months; OR, Odds Ratio; OS, Observational Study; patients, pts; placebo, plb; RCT, Random-Controlled Trial; SCORAD, SCORing Atopic Dermatitis; TIS, Three Item Severity score; Th, T helper; y, years; wks, weeks.

## 3 Oxidative stress in atopic dermatitis

**Figure 1:**
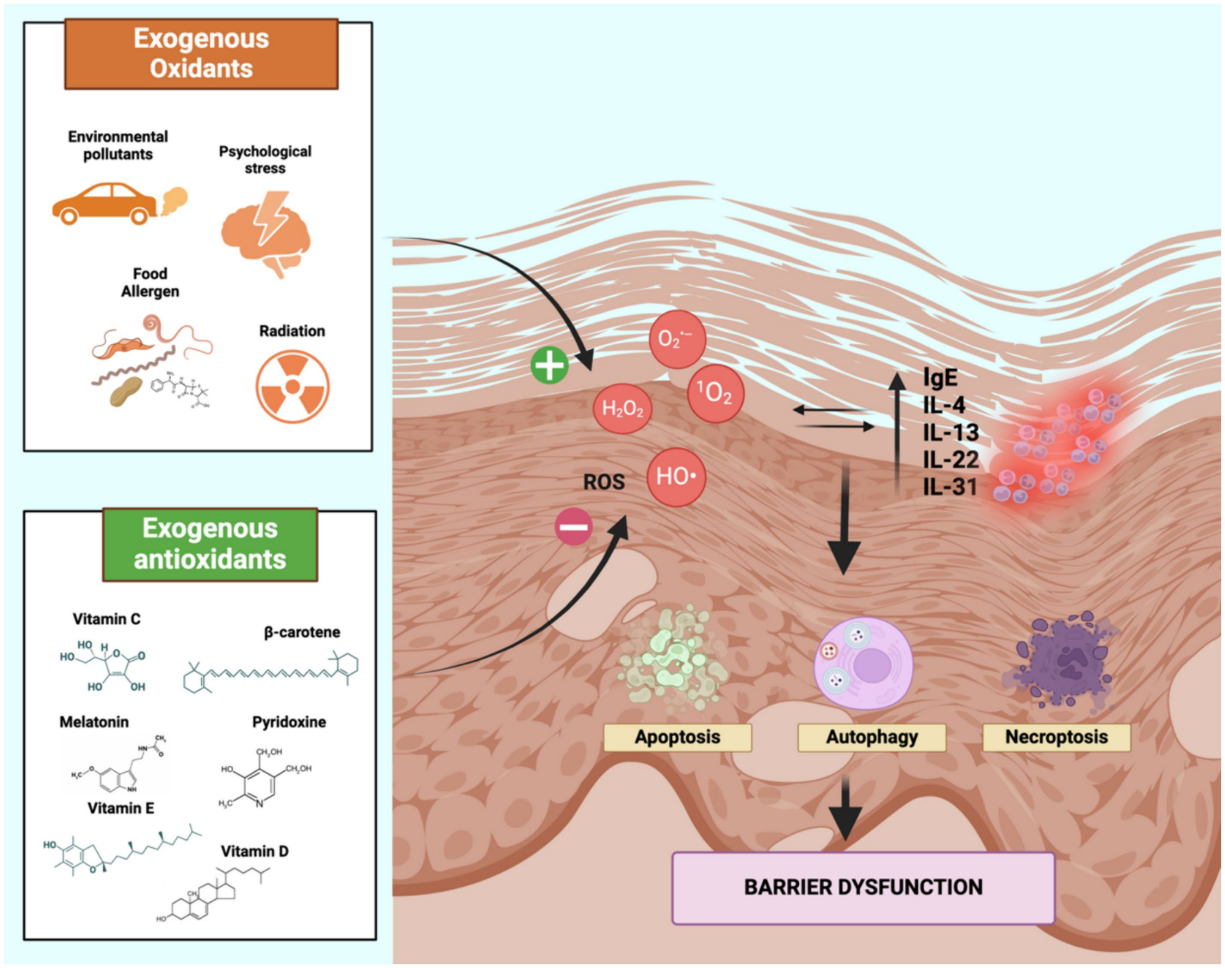
Oxidative stress-mediated inflammatory pathway in AD. Oxidative stress causes direct damage to cell membranes by the activation of programmed cell death mechanims, resulting in the disruption of the skin barrier. As a result, inflammatory cytokines including IL-4, IL-13, IL-22, IL-31 and IgE are released, leading to further ROS production, establishing a vicious cycle that underlies the pathogenesis of AD. Therefore, the balance between oxidants and antioxidants may avoid the overproduction of ROS in the skin and is influenced by environmental factors (exogenous oxidants and antioxidants). Created with Biorender.com. IL, Interleukin; ROS, reactive oxygen species.

ROS, including singlet oxygen (^1^O_2_), superoxide anion (O_2_^•–^), hydroxyl radical (HO•) and hydrogen peroxide (H_2_O_2_), can cause cellular damage and have been implicated in various diseases such as psoriasis, cancer, aging, atherosclerosis (24, 43–45). Elevated levels of total immunoglobulin E (IgE) and pro-inflammatory cytokines such as interleukin (IL)-4, IL-13, IL-22 and IL-31 can increase ROS production and vice versa, further contributing to the pathophysiology of AD (20, 24). The high production of ROS can induce autophagy, apoptosis and programmed necrosis in cells, resulting in DNA damage and skin barrier breakdown. In particular, the activation of mitogen-activated protein kinase (MAPk) pathway (especially of extracellular-signal-regulated kinase (ERK) and p38) and the inhibition of Mammalian target of rapamycin (mTOR) activity promote autophagy. In addition, intracellular oxidative damage can activate apoptosis via the C-Jun N-terminal kinase (JNK) pathway, a MAPk that activates p53. Furthermore, programmed necrosis can also be influenced by ROS, which promote the formation of a stable association between two serine/threonine kinases, namely RIP1 and RIP3, which mediate the complex formation of programmed necrosis (Figure 1) (22, 24, 46, 47).

The AD inflammatory response involves both adaptive and non-adaptive immunity and occurs in two-steps (1, 48). In the acute phase, the allergens cross the immune barrier and stimulate a T helper (Th) 2 response with the production of IL-4, IL-13, IL-22 and IL-31. Mast cells (MCs) are stimulated and degranulate, releasing histamine, IL-6, IL-8, prostaglandin D2 and tumor necrosis factor-alpha (TNF)- α together with IL-23 and IL-31 and other cytokines (48, 49). Additionally, keratinocytes and Langerhans cells (LCs), release cytokines such as IL-10, IL-12, IL-18, IL-23, IL-17 and IL-22 and thymic stromal lymphopoietin (TSLP), leading to an enhanced Th2-type inflammatory response (48, 50, 51).

The production of IL-2 and IL-18 by inflammatory epidermal dendritic cells (IDEC) or eosinophils and the subsequent activation of Th1 cells facilitate the transition to the chronic inflammatory phase with the production of TNFα, IL-2, IL-12, INFα (52). Interferon-gamma (IFN-γ) released by Th1 cells induces keratinocyte apoptosis, while IL-22 promotes skin changes and thickening in chronic AD. Chronic skin lesions in AD are associated with increased release of IL-5 and IL-12 and decreased levels of IL-4 and IL-13 (48). In addition, there is an increase in lymphocyte differentiation in the direction of Th 22 and Th 17, leading to the production of Il-22 and Il-17, respectively. The latter is involved in the altered proliferation and differentiation of keratinocytes (52).

Angiogenesis, the process of forming new blood vessels, contributes to the pathophysiology of AD and other diseases such as psoriasis and melanoma (53–55). Keratinocytes and mast cells have been shown to be a major source of vascular endothelial growth factor (VEGF) expression in AD; in fact, VEGF levels are elevated in the serum and skin of patients with AD compared with healthy controls (53–57).

ROS play a crucial role in the pathogenesis of AD because, by reacting with all macromolecules (lipids, proteins, nucleic acids, and carbohydrates), they promote a chain reaction that leads to damage and cell death. In addition, they cause the upregulation of genes coding for proinflammatory cytokines, thus contributing to the initiation of a vicious cycle that is self-feeding (47).

The human body responds to oxidative damage with an antioxidant defense system, including enzymes such as superoxide dismutase (SOD), glutathione peroxidase (GPx), catalase (CAT) and glutathione reductase (GR), as well as vitamin C, vitamin E, Vitamin D, carotenoids and glutathione (GSH). The skin is characterized by an endogenous antioxidant system which constantly counteracts the aggression of exogenous oxidants from the external environment. The epidermis, especially the stratum corneum, has a higher antioxidant capacity and acts as the body’s primary defense against both environmental and endogenous pro-oxidants. ROS generated at physiological levels during normal metabolism cause minimal damage to the skin due to the counterbalance provided by antioxidant species. However, excessive levels of ROS can overwhelm the skin’s antioxidant defenses and potentially contribute to the development of inflammatory skin disorders (24).

In particular, both superoxide dismutase (SOD) and catalase (CAT) play crucial antioxidant roles in the epidermis, showing a gradient of activity that decreases toward the skin surface. Interestingly, SOD activity is not affected by continuous sun exposure. However, it has been observed that SOD levels and activity decrease when the skin is exposed to greater amounts of sunlight. This response has been associated with exposure to ultraviolet A (UVA), while ultraviolet B (UVB) does not appear to have a similar effect. However, the skin demonstrates the ability to restore normal CAT activity within a period of 4 weeks after exposure, a period influenced by the patient’s age (39, 40).

Oxidative stress and lipid peroxidation contribute to the progression of inflammation in AD, as well as psoriasis, resulting in decreased antioxidant enzyme activities and levels of non-enzymatic antioxidants (45, 58, 59).

Among antioxidant enzymes, our recent studies have shown evidence of impaired functioning of the enzyme paraoxonase 1 (PON1) associated to high-density lipoprotein (HDL) in individuals with AD, with reduced protein levels and enzyme activity (60, 61). The elevated ratio of serum myeloperoxidase (MPO) to PON1 levels in AD patients confirms that HDL are dysfunctional in AD patients (62, 63). The presence of oxidized phospholipids and lysophospholipids, together with pro-inflammatory proteins, in dysfunctional HDLs, obstacles cholesterol efflux, resulting in LDL oxidation. This impairment of HDL function may contribute to lipid peroxidation of plasma lipoproteins, thereby fueling inflammation and promoting disease progression (62, 64). Additional research has demonstrated alterations in HDL function in AD with increased agonist-induced eosinophil effector responses compared to control patients (65, 66). While the precise role of ROS in the pathophysiology of AD has yet to be fully understood, numerous studies have shown that dietary factors which behave as antioxidants, modulate oxidative stress and gene expression of antioxidant enzymes.

In this regard, Klisic A. et al., assessed serum redox homeostasis in patients with psoriasis and AD in order to identify a specific oxidative stress biomarker for these diseases. There were no significant differences in oxidative stress biomarkers between the psoriasis group and the AD group, except for increased CAT activity in the AD group (*p* < 0.001), an enzyme which contributes to neutralization of ROS-mediated damaging effects by converting hydrogen peroxide into water and oxygen (67).

Moreover, epidemiological studies of adults with AD provide strong evidence about its association with obesity. Not only does it represent a risk factor for AD, but also it contributes to increased inflammation. In obese patients, the expansion of adipose tissue can stretch the epidermis, compromising its barrier function and triggering the innate immune cascade underlying the autoimmune process. The skin barrier defects typical of obesity can facilitate the penetration of allergens and/or pathogens through the skin. This promotes the development of a Th2-type immune response and subsequent acute allergic inflammation: both contribute to AD pathogenesis. Conversely, patients with severe AD show a greater predisposition to obesity, partly due to a more sedentary lifestyle (10, 68–70). Dietary treatment with antioxidant compounds can yield improvements in AD patients by maintaining ROS levels within appropriate physiological limits.

## 4 Antioxidant agents

### 4.1 Vitamin D

Vitamin D is a fat-soluble vitamin involved in the regulation of calcium-phosphorus metabolism, immune system activity and inflammatory processes (71). Indeed, vitamin D has been shown to upregulate the expression of various antioxidant enzymes, such as SOD, thioredoxin reductase (TrxRs). This gene overexpression mediated by vitamin D has been observed in prostate cells, where it has demonstrated a protective effect against cell death induced by H_2_O_2_ (47).

In the skin, vitamin D regulates the production of antimicrobial peptides such as cathelicidin and human beta-defensin (HBD) by skin keratinocytes and monocytes but its antioxidant activity in skin cells needs to be fully elucidated. The antimicrobial peptides contribute to prevent skin infections and participate in the synthesis of filaggrin, an important component of the skin barrier (71, 72). Vitamin D suppresses the differentiation of dendritic cells and the proliferation of Th1 lymphocytes by reducing the secretion of Th1 cytokines. It also modulates the secretion of cytokines Th17 and IL-2 by regulatory T cells (Treg) (71). The body’s need for vitamin D can be met by exposure to the sun and dietary sources such as fish, egg yolk, and dairy products.

*In vitro* studies and clinical trials have demonstrated the role of vitamin D in AD, although the findings are not yet entirely conclusive (73). Controversial data have been reported on the relationship between oral vitamin D supplementation and AD severity. Most studies have found an inverse relationship between vitamin D levels and the onset and severity of AD; the most recent studies are reviewed below.

In a study conducted in a pediatric population, a significant reduction in EASI was found among patients treated with vitamin D, compared to those in the placebo cohort. Subjects were randomly assigned to receive either a daily dose of 1,600 IU vitamin D or a placebo. This regimen was administered together with a baseline therapy consisting of topical application of a 1% hydrocortisone cream twice daily for a duration of 12 weeks. The mean EASI score in the treatment group showed a significant reduction compared to the placebo group (*p* = 0.035). In addition, there was a significant improvement in EASI score from baseline between the supplementation group (56.44 ± 29.33) and the placebo group (42.09 ± 19.22) after treatment (*p* = 0.039) (32) (Table 1).

A study by Peroni et al. investigated the correlation between reduced serum vitamin D levels and the severity of AD in 37 pediatric patients, age range 8 months to 12 years. Levels of vitamin D, determined by the chemiluminescence method, were significantly higher in patients with mild disease than in those with moderate or severe disease (74). Furthermore, in a prospective observational study conducted by Tsotra et al. 50 children were enrolled, and a statistically significant reduction in SCORAD was observed after 20 weeks of treatment in children who had received 2,400 IU of vitamin D. Specifying that the reduction becomes nonstatistically significant after 45 days and at the end of treatment (75).

Similarly, in open-label supplementation study, 22 children with AD were enrolled and received weekly oral vitamin D for 6 weeks, improvement in AD was observed with a reduction in SCORAD from 41.4 ± 13.5 to 31.5 ± 15.8 (*p* < 0.0001) and an increase in cathelicidin expression on skin lesions (*p* < 0.0001) (34).

However, other studies have shown conflicting results. A cross-sectional study was conducted in 77 patients with AD under the age of 18. In the first phase, the relationship between vitamin D levels and disease severity was assessed, and in the second phase, 44 patients were randomized to receive vitamin D supplementation at 2000 IU/d or placebo. The results showed that disease severity was significantly associated with lower vitamin D levels (*p* = 0.015) and that the mean change (standard deviation) in severity was not significantly different between the supplementation (15.35 [9.71]) and placebo (15.13 [8.97]) groups after 3 months of intervention (*p* = 0.7) (31).

In the randomized study conducted by Galli et al., 89 children with AD were enrolled. One group received 2000 IU/day of Vitamin D, while another group received a placebo for a duration of 3 months. It was observed that vitamin D supplementation did not influence the SCORAD severity or the concentration of total IgE, and no statistically significant correlation was found between vitamin D levels and SCORAD at baseline (28).

In another trial, 65 patients were randomized to receive standard therapy (topical corticosteroids, emollients) with 5,000 IU/day of vitamin D or placebo, for 3 months. The achievement of serum levels of vitamin D of 20 ng/mL, in combination with standard treatment, was demonstrated to be sufficient to reduce SCORAD in patients with AD (30). Di Filippo et al. similarly administered oral vitamin D supplementation 1,000 IU/day (25 mg/day) to patients with AD. A statistically significant reduction in the SCORAD was observed after 3 months of supplementation (46.13 ± 15.68 at the baseline versus 22.57 ± 15.28 at the post-treatment assessment; *p* < 0.001). In addition, there was a reduction in IL-2, IL-4, IL-6 and IFN-γ compared to baseline (29). Similar results had previously emerged in an RCT including 60 patients, where an improvement in disease severity was observed in the group treated with 1,600 IU of vitamin D for 60 days, compared with the control group (26).

The association between vitamin D supplementation and increased levels of cathelicidin and HBD has also been questioned. In a double-blind study, 30 AD patients, 30 non-atopic subjects and 16 patients with psoriasis were randomized to receive vitamin D 4000 IU or placebo for 21 days. The levels of vitamin D, cathelicidin, HBD, IL13 and the EASI were measured. After 21 days, serum levels of vitamin D had increased, but there were no significant changes in skin levels of cathelicidin, HBD, IL-13 or the EASI. Low levels of vitamin D correlate with increased skin pigmentation and body mass index (BMI), but not with AD severity (27).

However, these studies are limitated by the inability to control for sun exposure, dietary vitamin D intake, basic therapy for AD, and the lack of longitudinal follow-up (27, 35, 76, 77). It is also important to analyze baseline levels of vitamin D in the different patients being studied because differences in vitamin D levels at baseline may confound the results of the trials.

As far as vitamin D supplementation during pregnancy, the MAVIDOS double-blind randomized controlled trial examined the association between the administration of 1,000 IU vitamin D or placebo daily from the 14th week of gestation until delivery, and AD severity in offspring at 12, 24, and 48 months of age. Offspring AD prevalence was assessed at 12 (*n* = 635), 24 (*n* = 610), and 48 (*n* = 449) months of age. In the treatment group, compared to control, at 12 months AD prevalence was lower (OR 0.55; *p* = 0.04); this effect attenuated and was not statistically significant at 24 months (OR 0.76) or 48 months (OR 0.75) (33).

In conclusion, a meta-analysis included 20 studies with 1882 AD cases showed an association between low vitamin D levels and AD compared to healthy controls (*p* < 0.001). In addition, significantly lower vitamin D levels were observed in severe AD compared to mild-to-moderate AD (*p* < 0.001). This relationship was further supported by the improvement in AD symptoms in patients receiving vitamin D supplementation (*p* < 0.001) (78). Moreover, contrasting results emerged in an other meta-analysis in which 5 RCTs with 304 AD cases were included. Vitamin D supplementation did not lead to a reduction in AD severity in studies that included only children, but reduced AD severity in those with heterogeneous populations and in those with a vitamin D dose >2,000 IU/day (79).

### 4.2 Vitamin E

Vitamin E is an essential nutrient with antioxidant properties and includes both tocopherol (TP) and tocotrienol (T3), differing in their aliphatic tails (80). It serves as crucial antioxidant barrier in human skin and is implicated in suppressing inflammation and promoting keratinocyte differentiation. The interest to investigate the role of Vitamin E in AD is supported by lower levels of vitamin E in AD patients (81, 82).

Preclinical studies have described the role of Vitamin E in AD: it seems to reduce transepidermal water loss (TEWL), which is associated with AD pathogenesis, and to induce the expression of the transglutaminase-1 gene, involved in terminal differentiation of keratinocytes and in the formation of the stratum corneum (83–85).

Case–control studies highlighted an inverse association between vitamin E and AD. In a pediatric study of involving 180 children with AD and 242 healthy controls, vitamin E levels were found to be inversely associated with AD prevalence (23). Similar results were reported in studies conducted with Japanese students and Korean children, where higher dietary intake of vitamin E or higher serum alpha-TP levels were associated with improved AD symptoms and lower serum total IgE levels (36, 86).

A study assessed the influence of maternal diet during pregnancy in a population comprising 557 mother–child pairs and showed that inadequate vitamin E intake during pregnancy was correlated with an increased risk of AD (OR = 2.7, *p* = 0.04) (87). Supplementation with vitamin E and/or vitamin D improved the clinical manifestations of AD, as evidenced by a reduction in the SCORAD, and also led to an increase in SOD and catalase activity (35, 88).

In a double-blind, randomized, placebo-controlled study, Jaffary et al. explored the correlation between oral vitamin E and AD. They enrolled 70 patients with AD who received either a daily dose of 400 IU of vitamin E or a placebo for a duration of 4 months. The results of this study indicate that vitamin E is effective in relieving itching in patients with AD (37). However, it is worth noting that in the research conducted by Daniluk et al. no significant changes in tocopherol levels were observed when comparing the group of AD patients with the healthy control (89).

In conclusion, in a meta-analysis of four studies, which included 259 AD patients and 307 healthy controls, it was observed that AD patients had significantly lower serum vitamin E levels than the control group (standardized mean difference: -1.08, 95% CI: −1.80 to −0.36) (81).

### 4.3 Vitamin C

Vitamin C or ascorbic acid is an essential micronutrient with pleiotropic functions. It acts as a potent antioxidant and serves as a cofactor for regulatory enzymes. Since humans cannot synthesize vitamin C, it must be obtained exclusively from the diet, mainly from fruit and vegetables (90). In the context of AD, the abnormal immune response leads to elevated levels of cytokines such as IL-2, resulting in increased production of ROS and lipid peroxidation (91). As an antioxidant, vitamin C has a crucial role and protects against oxidative stress-induced cellular damage by scavenging of ROS. Moreover, vitamin C promotes keratinocyte differentiation and enhances the production of interstitial material, in order to maintain the normal function of the skin barrier (90). The antioxidant role of vitamin C may explain the observed low plasma levels of vitamin C in adults with AD. Additionally, vitamin C levels in the dermis of patients with AD are significantly lower compared to those of healthy individuals (38, 91).

In a study recruited breastfeeding women with AD. They were subjected to a diet enriched with vitamins C and E, with the aim of examining the composition of breast milk and investigating whether this dietary intervention had a protective effect against the development of atopy in newborns. Neonatal atopy was defined as the presence of AD during the first year of the child’s life. It was observed that a higher concentration of vitamin C in breast milk was strongly associated with a reduced risk of atopy in newborns (OR = 0.30; 95% CI 0.09–0.94; *p* = 0.038), while α-tocopherol showed no significant relationship with atopy (92). Another study involving 17 patients with AD, 82.4% of them had plasma vitamin C levels below 25 μmol/L, while only 3 patients had levels within the normal range (25–80 μmol/L). Notably, two patients with severe AD exhibited particularly low vitamin C levels of 8.24 and 12.30 μmol/L. These findings suggest a correlation between the progressive worsening of moderate–severe AD and vitamin C decreased levels (93).

Despite, its recognized benefits as an adjuvant therapy for various inflammatory skin conditions, the inclusion of vitamin C in cosmetic creams may potentially induce symmetric AD (70, 73).

To better evaluate this correlation and explore the potential therapeutic role of vitamin C, further large-scale data are needed on skin levels of vitamin C in patients with moderate and severe AD.

### 4.4 Pyridoxine or vitamin B6

Pyridoxine or vitamin B6, is an essential water-soluble vitamin involved as cofactor in different metabolic pathways (94). These pathways include catalyze transamination, decarboxylation and protein synthesis, carbohydrate metabolism, nucleic acid transcription, glucocorticoid receptor regulation and neurotransmitter synthesis (38, 95).

In a recent study that included 48 patients 19 were treated with 50 mg/day pyridoxine hydrochloride and 22 with placebo, no statistically significant differences were found between the two groups (72).

The active form of vitamin B6, known as pyridoxal 5′-phosphate (PLP), plays a crucial role as a co-factor in over 150 enzymatic reactions. There is evidence of consistently low plasma PLP levels in inflammatory conditions. Therefore, intake and supplementation of vitamin B6 are beneficial for improving certain immune functions, both in humans and in vitamin B6-deficient laboratory animals (96).

In a study, the silico analysis of gene expression data and analysis of serum vitamin B6 metabolites in patients with psoriasis indicated altered vitamin B6 metabolism both locally and systemically. Functional studies have shown that vitamin B6 reduces neutrophil infiltration into the skin, oxidative stress and NF-kB activity in two models of skin inflammation. Strikingly, inhibition of glycogen phosphorylase L (Pygl) and glucose-6-phosphate dehydrogenase (G6pd), two enzymes regulated by vitamin B6, attenuated oxidative stress-induced inflammation in zebrafish models of skin inflammation (97).

### 4.5 Melatonin

Melatonin is an indolamine hormone primarily produced by the pineal gland, although it can also be synthesized by other tissues such as the thymus, respiratory epithelium and bone marrow (98). It is a potent endogenous antioxidant and exhibits strong anti-inflammatory properties, as demonstrated by different *in vivo* and *in vitro* studies (99–101). Therefore, some authors have investigated the effect of melatonin intake as supplement on biomarkers of oxidative stress and antioxidant enzymes. Melatonin stimulates key antioxidant enzymes like SOD, GPx and GR, protecting cells from lipid peroxidation and neutralizing harmful radicals. In addition to its antioxidant and anti-inflammatory effects, melatonin plays a crucial role in regulating the circadian rhythm. Melatonin receptors, specifically MT1 and MT2, are expressed in the skin (102).

In AD, melatonin is hypothesized to preserve skin integrity and maintain a functional skin barrier through its antioxidant properties, which include reducing lipid peroxidation and exerting anti-apoptotic effects (103, 104). Patients with AD often exhibit reduced production of IFN-γ, leading to decreased melatonin production. This, in turn, results in sleep deprivation and additional stress for patients with AD (105, 106).

Preclinical data also suggest that melatonin, as well as its precursor 1-tryptophan, can reduce IgE and IL-4 levels, preventing the development of AD (107). In addition, melatonin appears to hinder the activation, degranulation and infiltration of mast cells, which are involved in skin damage (99). Munoz-Hoyos et al. showed that during AD onset phase, patients had reduced circulating levels of melatonin and beta-endorphin, despite higher nocturnal levels of both hormones. Their hypothesis is that the physiological peak of melatonin produced at night may compensate for the decreased melatonin production by extra-pineal tissues during the daytime, which occurs during AD onset episodes (108).

A study on 72 children with AD also confirmed a lower nocturnal secretion of melatonin in patients with AD. In addition, a reduction in sleep quality was observed in terms of longer sleep onset latency, more sleep fragmentation and less non-rapid eye movement sleep (109).

In a randomized clinical trial including 73 children aged between 1 and 18 years, with at least 5% skin involvement by AD, compared melatonin (3 mg/day) with placebo, both administered for a 4-week period, with a 2-week washout period. Following melatonin treatment, the SCORAD decreased by 9.1 points compared to placebo (from 49.1 to 40.2). Moreover, the latency period for sleep was reduced by 21.4 min after melatonin treatment compared to placebo. However, it should be noted that the improvement in SCORAD did not exhibit significant correlation with changing sleep latency (39). Furthermore, in a randomized, double-blind, placebo-controlled study enrolling 70 patients with AD aged 6 to 12 years, the administration of melatonin (6 mg daily) demonstrated to improve SCORAD (*p* = 0.007), objective SCORAD (*p* = 0.001), serum total IgE levels (*p* = 0.005) and Children’s Sleep Habits Questionnaire scores (*p* = 0.006) at 6 weeks (40).

In contrast, a study on 30 patients with AD aged 6 to 12 years and 30 healthy individuals evaluated serum levels of melatonin, SOD, and GPx and showed that melatonin levels were higher in AD group compared to the control group. However, it should be noted that the levels of GPx and SOD were also higher in AD cases, although the observed difference was not statistically significant (110).

### 4.6 Carotenoids

Carotenoids are powerful antioxidant compounds involved in ROS scavenging and in protection of skin barrier integrity. The plasma/serum levels of total carotenoid content is a reliable indicator of the body’s overall antioxidant status. The skin predominantly contains α-, γ-, β-carotene lutein, zeaxanthin, lycopene, and their isomers as the main forms of carotenoids (111, 112). The human body cannot synthesize carotenoids. Therefore, dietary intake is essential to fulfill the body’s carotenoid requirements. After digestion, carotenoids are absorbed and exert their beneficial effects. Excess carotenoids can be stored in fatty tissue, liver, and other organs (113, 114). Vegetables and fruits are the primary dietary sources of carotenoids, responsible for the red, orange, and yellow colors found in nature (115, 116). They can also be found in certain animals such as fish and crustaceans, although to a lesser extent (115).

Carotenoids are also utilized in dermatological and cosmetic products as active ingredients for skin application (114). The biological functions of carotenoids are closely related to their chemical structure. Most carotenoid molecules have a characteristic skeleton of 40 hydrocarbons with a system of conjugated double bonds. This structure is crucial for light absorption in photosynthetic organisms and photoprotection ROS generated by sunlight (115, 117).

*In vitro* and *in vivo* tests have revealed the effects of carotenoids on patients with AD (117).

Some studies highlighted the role of vitamin A deficiency in the pathophysiology of AD, especially in early childhood. Timely vitamin A supplementation during pregnancy and childhood could prevent the onset or exacerbation of AD (118).

A study by Rühl et al. reported significantly lower serum vitamin A concentrations in infants with AD. Specifically, lower concentrations of lycopene and non-vitamin A carotenoids were observed in atopic infants, suggesting that lycopene may provide protection against atopy (119). Regarding the intake of antioxidant nutrients and their protective effect against AD, it has been found that β-carotene can reduce the risk of AD. Children with a higher intake of β-carotene have a lower risk of developing AD (OR = 0.69, *p* = 0.0166) (23).

Maternal consumption of vegetables during pregnancy has also been associated with a lower likelihood of eczema and asthma (120). A prospective study by Inoue et al. on a cohort of 267 infants with a family history of allergy found that certain levels of carotenoids, such as lutein and lycopene, were associated with a reduced likelihood of developing AD at 1 year of age (*p* < 0.001) (42). Moreover, in a pediatric study, the correlation between vitamin A and D deficiency and the severity AD was assessed. The results revealed that serum levels of both vitamin D and A were significantly lower in children with AD compared to healthy children. Additionally, the concurrent deficiency of both vitamins was linked to a greater severity of AD (41).

## 5 Discussion

Skin barrier dysfunction is one of the main hallmarks of atopic dermatitis and is responsible for increased access of environmental agents to the skin. These promote an increase in ROS, thereby triggering an inflammatory immune response. The importance of ROS may be particularly relevant in extrinsic atopic dermatitis, characterized by continuous skin barrier dysfunction. In this regard, the contribution of antioxidant substances may be of greater benefit in this category of patients, especially in the early stages of the disease.

Oxidative stress consists of an excess production of ROS caused by a disturbance in oxidative homeostasis. The surplus of ROS represents a trigger for programmed cell death processes and promotes the release of pro-inflammatory cytokines, which are also responsible for further increasing ROS levels, thus perpetuating the pathophysiological process of AD.

Both exogenous and endogenous antioxidants can manipulate the ROS levels, by regulating gene expression and signaling pathways, and eventually maintain the oxidative balance and the integrity of cellular components (24, 121).

The onset and the pathophysiology of AD are characterized by oxidative stress and inflammation. Clinical evidence suggests a possible role for antioxidant agents as adjunctive therapy for patients with AD and in its prevention. They could be employed to reduce the use of steroids, thus helping to mitigate the adverse events associated with them (122).

Data from the studies analyzed suggested a potential role for several antioxidant molecules including vitamin D, vitamin E, vitamin A and melatonin. The most promising role seems to be played by vitamin D, with several studies indicating its possible therapeutic use in AD (26, 30, 32, 75). In particular, there is one RCT study conducted on 60 patients in which significant improvement was observed in patients treated with 1,600 IU of vitamin D for 60 days. However, even for vitamin D, not all studies offer concordant results (26). In fact, another study conducted on 77 patients for 1 day showed no difference between patients treated with vitamin D 2000 IU and placebo (31). Less evidence is available for vitamin E, however, with only one randomized study highlighting its potential role in the treatment of the condition (35).

Similarly, some randomized studies suggest a potential benefit in the administration of melatonin at variable doses (3 to 6 mg/day) with a significant reduction in SCORAD in both cases (39, 40). Therefore, the antioxidant species were used at different doses, so further clinical studies are needed to identify the minimum useful dose to be used as monotherapy or in combination with conventional therapies.

However, the role of the age and the efficacy of combination approaches have to be further assessed. Indeed, existing evidence suggests that antioxidants are more effective in children than in adults, which could be explained by a different maturational state of the immune system and a different prevalence of inflammatory cytokines in AD pathogenesis. Furthermore, a meta-analysis reported an improvement in AD severity in patients who received a combination of antioxidants, as demonstrated for oral vitamin D and topical vitamin B12, especially in pediatric patients (123).

Additional data come from studies on other immune disorders such as psoriasis, lichen planus, and vitiligo, where ROS contribute to the pathophysiology of the disease. In this setting as well, treatments with antioxidant substances could be appropriate, but literature data indicate that antioxidants alone are unable to induce significant clinical changes, except perhaps in mild forms, and must be used in conjunction with standard drug treatments to achieve measurable results (124).

Another aspect to be investigated is the combination of several antioxidant species to take advantage of the synergistic effect between them. In this regard, vitamin E is known to act synergistically with vitamin C to regenerate tocopheryl radicals, the oxidation product of alpha-tocopherol (125). Again, further studies would be needed to examine the synergy with traditional therapies used in AD, as synergistic effects between some antibiotic therapies and vitamin E derivatives involved in various infections are already known (126, 127).

## 6 Conclusion

In conclusion, oxidative stress is involved in AD pathophysiology, therefore there is a strong preclinical rationale to incorporate antioxidative agents, as adjuvant treatment, in the AD pharmacopeia. However, the available evidence is limited and heterogeneous. Therefore, well-designed RCTs with larger sample sizes are needed to evaluate the adjuvant role of antioxidants in combination with active treatments, especially with new biologic agents.

Pending this data, antioxidants should be considered on a case-by-case basis in patients with AD according to disease activity and severity, comorbidities, patient preferences, and drug interactions. In this regard, collaboration between nutritionists and dermatologists is needed to ensure optimal and safe use of exogenous antioxidant agents and, especially, to promote lifestyle modifications, including dietary, physical, and psychological changes.

## Author contributions

ES: Writing – original draft. MC: Data curation, Writing – review & editing. SB: Methodology, Writing – review & editing. GR: Data curation, Writing – review & editing. EM: Formal analysis, Writing – review & editing. IC: Validation, Writing – review & editing. GF: Writing – review & editing. TB: Writing – review & editing. AO: Resources, Writing – review & editing. OS: Writing – review & editing, Conceptualization, Supervision, Validation.
